# Does Discrimination Discriminate by Age and Zip Code? Area-Level Effects Among Black Women

**DOI:** 10.1093/geroni/igaf122.2121

**Published:** 2025-12-31

**Authors:** Brionna Colson-Fearon, H Shellae Versey

**Affiliations:** Fordham University, New York, New York, United States; Fordham University, New York, New York, United States

## Abstract

Black women experience everyday discrimination (ED) across multiple levels and social positionalities (e.g., race, gender, and gendered racism). Yet research exploring how and whether age intensifies the effects of ED is limited. Moreover, prior literature suggests that reports of discrimination may vary according to spatial indices (i.e., neighborhood racial composition, segregation). Among a national sample of Black women (N = 944) aged 18-80, we examine whether reports of ED frequency vary by age and place. We also explore whether area-level ethnic-racial similarity (e.g., neighborhood-level segregation) plays a role in reporting frequency. That is, does the density of Black residents within an area (e.g., higher percentage of Black racial composition) moderate this association? Using hierarchical linear models and data from the Our Story, Our Voices Survey of Black Women collected between 2019-2020, we find significant associations between age and ED where older Black women generally report lower frequencies of discriminatory experiences, compared to younger Black women. Findings regarding area-level effects are mixed; it is unclear whether area-level density of majority Black residents functions as a buffer against frequency of ED. Results reveal future directions for research in intersectional and gerontological research. Implications for the growing diverse older adult population are discussed.

